# In-silico investigation towards the non-invasive optical detection of blood lactate

**DOI:** 10.1038/s41598-021-92803-x

**Published:** 2021-07-12

**Authors:** Subhasri Chatterjee, Karthik Budidha, Meha Qassem, Panicos A. Kyriacou

**Affiliations:** Research Centre for Biomedical Engineering, City, University of London, London, EC1V0HB UK

**Keywords:** Engineering, Optics and photonics

## Abstract

This paper uses Monte Carlo simulations to investigate the interaction of short-wave infrared (SWIR) light with vascular tissue as a step toward the development of a non-invasive optical sensor for measuring blood lactate in humans. The primary focus of this work was to determine the optimal source-detector separation, penetration depth of light at SWIR wavelengths in tissue, and the optimal light power required for reliable detection of lactate. The investigation also focused on determining the non-linear variations in absorbance of lactate at a few select SWIR wavelengths. SWIR photons only penetrated 1.3 mm and did not travel beyond the hypodermal fat layer. The maximum output power was only 2.51% of the input power, demonstrating the need for a highly sensitive detection system. Simulations optimized a source-detector separation of 1 mm at 1684 nm for accurate measurement of lactate in blood.

## Introduction

Lactate is a metabolite of glucose produced by the human body during anaerobic energy production. It is transported from the body’s tissues to the liver by blood and is either oxidized into carbon dioxide and water or is converted to glucose in a cyclic process. However, this process requires adequate oxygen, and in conditions of hypoperfusion or hypoxia resulting from a severe infection, heart failure or respiratory failure, lactate clearance is hindered, leading to a build-up of lactate in the blood beyond the normal level (< 2 mmol/L). This condition, known as lactic acidosis can upset the body’s pH balance and often manifests life-threatening symptoms such as difficulty in breathing, confusion, and even coma^1^. Hence a continuous measure of blood lactate is essential in today’s critical care for early identification of acute conditions such as sepsis, heart failure, renal failure, severe inflammatory response syndrome, etc.

The prevalence of blood lactate in identifying poor prognosis in acutely ill patients is well known and has been the subject of research for many years. Yet there exists no tool to continuously measure blood lactate reliably and non-invasively in the clinical setting. The state-of-the-art remains to be intermittent measurements using arterial blood gas analyzers, which are invasive, costly, and complex to operate. Hence, there exists an unmet clinical need for a reliable tool that can measure blood lactate non-invasively in the clinical setting.

As a step towards the development of such a device, researchers have investigated the feasibility of using short-wave infrared (SWIR) light (typically, between the range of 1300–2500 nm) to detect the optical signature of lactate in blood and estimated the concentration of lactate from the acquired spectra using various regression models^2–8^. These preliminary in-vitro investigations have shown great promise and have paved the way for the development of a novel optical sensor for the blood-lactate measurements. However, these in-vitro experiments are unable to provide any information on the light-tissue interactions underlying the technique, which is key in assessing the efficacy of the sensor design for the lactate measurement. The applicability of a sensor largely depends on the anatomy of the tissue region-of-interest (ROI), and the design of the sensor (e.g., wavelength, shape and size of the optical source and detector, source-detector separations etc.). Such crucial details have so far never been considered. To address these issues, this paper aims to create a robust in-silico model based on preliminary in-vitro experiments, that can be easily modified to simulate any sensor-tissue interaction, thus assisting the development of a novel non-invasive optical sensor for blood lactate detection.

## Background

A number of in-silico and in-vitro models are available in the literature that may help design biophotonic sensors, however, those are mostly limited within the visible and near-infrared optical window. Applications in the short-wave infrared wavelength region remain largely unexplored which can be attributed to the fact that water is highly absorbing at this wavelength range making the analysis difficult to pursue. Recently, a few research works have investigated the SWIR light in vascular tissue with an interest in optical imaging and diagnosis^9–13^. Unfortunately, none of these models considered the presence of the lactate.

**Figure 1:**
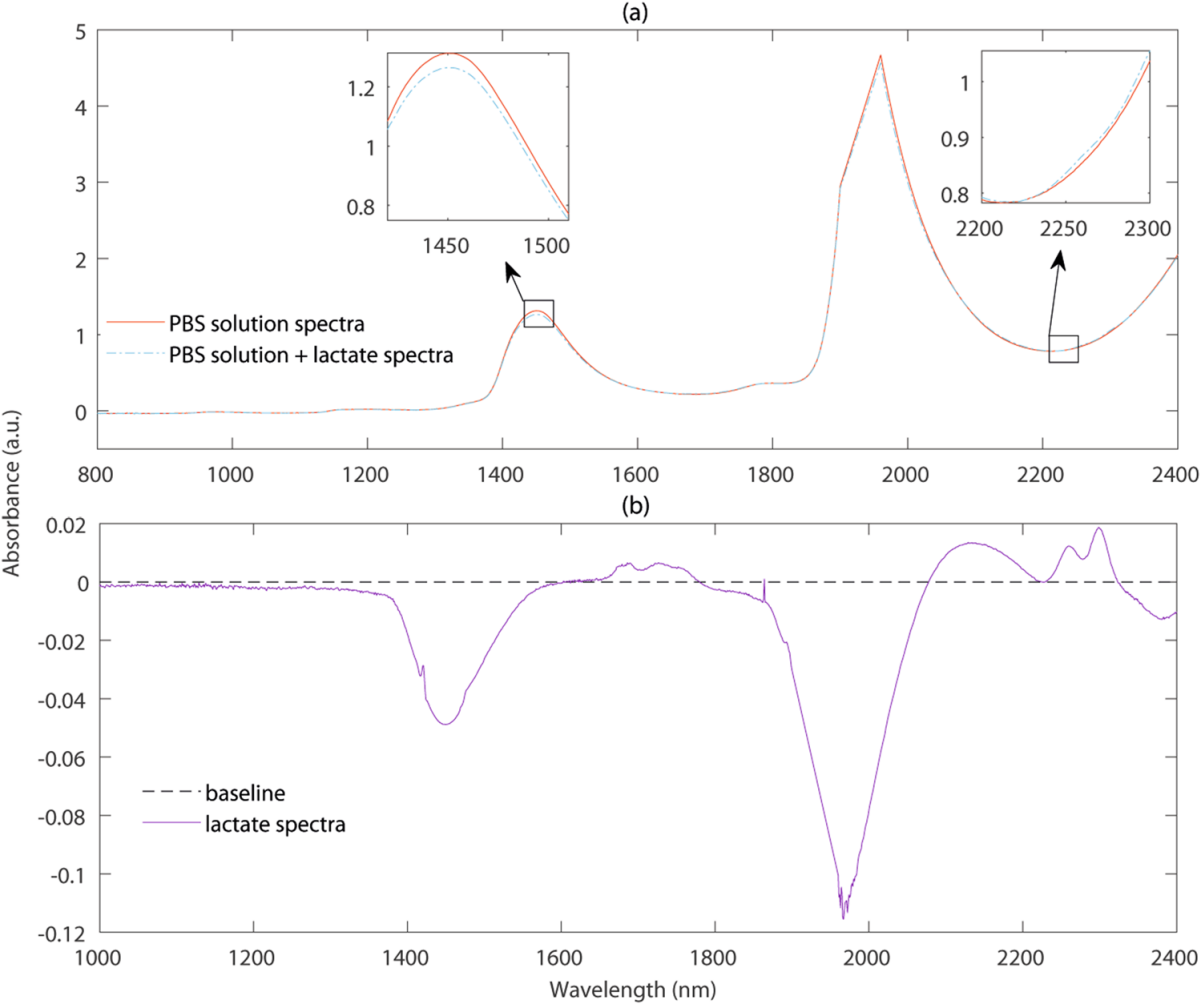
(**a**) The raw spectra of sodium l-lactate dissolved in aqueous PBS (concentration: 100 mmol/L) along with the spectra of just the aqueous PBS solution in the SWIR optical window. (**b**) Resultant spectra following the subtraction of aqueous PBS spectra from the lactate containing spectra.

As a part of our previous in-vitro investigation, we found that lactate absorbance peaks are detectable at the SWIR wavelengths even though water absorbance prevails^5^. Figure 1a shows the raw spectra (recorded using the Lambda 1050 dual-beam UV/Vis/NIR spectrophotometer, Perkin Elmer Corp, Massachusetts, USA) of sodium l-lactate (L14500, Alfa Aesar, Lancashire, UK) dissolved in aqueous PBS (concentration: 100 mmol/L) along with the spectra of an aqueous PBS sample. As can be seen from the figure, water is highly absorbing in the SWIR region, making it difficult to distinguish the lactate absorption from that of water. However, following the subtraction of PBS spectra (water peaks) from the lactate containing spectra as in Fig. 1b, the absorption of lactate and its peaks become evident. At the water peak wavelengths, for example, around 1450 nm and 2000 nm, PBS solution absorbance is higher compared to the lactate solution absorbance since water volume decreases in the sample as lactate is added to the solution. Therefore, the subtraction of water (or PBS solution) absorbance spectra from the lactate + PBS solution absorbance spectra results in negative peaks at those wavelengths as shown in Fig. 1b. On the other hand, at wavelengths such as 2259 nm where water absorbance is low, lactate + PBS solution absorbance is higher. Consequently, the subtraction results in a prominent lactate absorbance peak as shown in Fig. 1b.

Motivated by our initial findings, we have carried out the current in-silico investigation which could aid in the design of an optical sensor for blood-lactate detection. This investigation has been based on the Monte Carlo (MC) modelling method.

Monte Carlo models of light-tissue interactions have been extensively used for various bio-photonic applications including diffuse spectroscopy, optical coherence tomography (OCT), photoacoustic tomography, endoscopy and so on^14–18^. MC simulates the photon trajectory through the medium by random sampling of variables in terms of the absorption and scattering properties of the medium. Compared to other available tissue-optics models (such as diffusion theory, random walk model, finite element model etc.), MC exhibits several advantages such as its ability to produce accurate results at any source-detector separation, inclusiveness of any complexity of the tissue-structure, multiple random scattering events and any sensor geometry etc. MC, however, has a major drawback which is its intensive computation time. In order to overcome this limitation, several different approaches have been incorporated by researchers, for example, scaling methods^19^, perturbation methods^20^, hybrid methods^21–23^, mesh based modelling^24^, variance reduction techniques^25–27^ etc. With the recent significant advancements of computer technology, multi-threaded platforms and especially GPU-accelerated programming have made MC simulation remarkably faster^28–33^. The plethora of research on MC shows the credibility of this technique, and justifies it as the most suitable platform for the current in-silico investigation.

## In-silico modelling strategy

**Figure 2:**
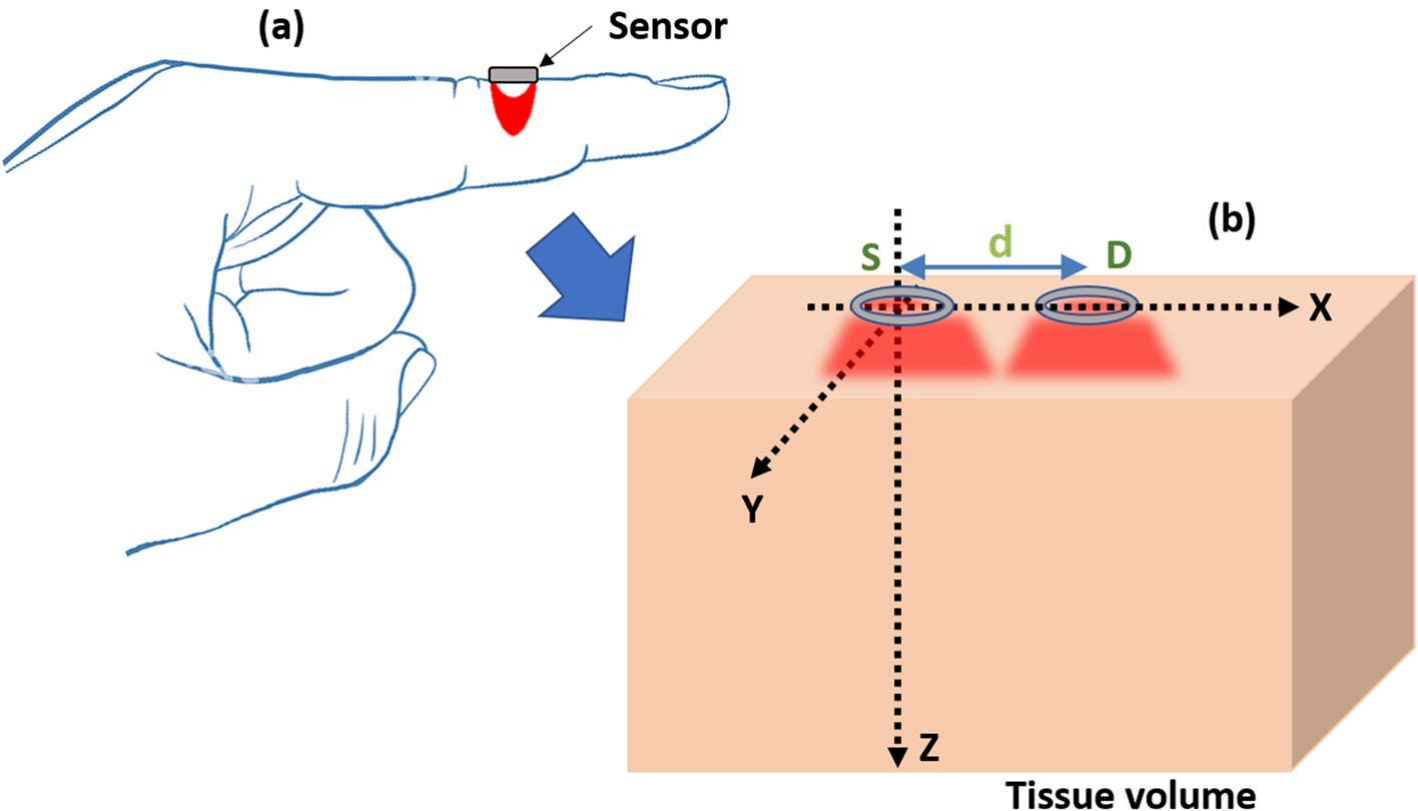
Schematic of the sensor-tissue interaction platform. The ROI, i.e., a volume of human finger tissue interrogated by the sensor, is presented in the 3D Cartesian co-ordinate (XYZ) where the optical source (S) and detector (D) are respectively placed at the origin, and a distance *d* from it.

A robust MC model of finger-ROI was developed and executed at a reflectance sensor geometry as shown in the schematic diagram in Fig. 2. The MC model was explored for determining: (1) the mean penetration depth of light at SWIR wavelengths in vascular tissue, (2) the optimal source-detector separation for the acquisition of lactate spectra and (3) the optimal light power required for reliable detection of lactate concentration in blood.

Whilst simulations at all continuous wavelengths as shown in Fig. 1 would produce a wide range of information, it was not achievable in a realistic time frame. Therefore, as an holistic and timely approach, *ten* discrete characterizing SWIR wavelengths were selected based on an observational assessment from Fig. 1. The wavelength selection was made in three steps:

- *Lactate peak wavelengths:* At 1684 nm, 1730 nm, 1752 nm, 2129 nm, 2259 nm and 2299 nm, prominent lactate peaks and weak water peaks are visible in Fig. 1. Therefore, these wavelengths are useful to detect the absorbance due to lactate.
- *Water peak wavelengths:* At 1920 nm, very strong water absorbance peak is visible in Fig. 1. This wavelength is useful to investigate the sensor performance where maximum light is absorbed in tissue.
- *Non-peak wavelengths:* At 1310 nm, 1550 nm and 1650 nm, no high peaks of water or lactate is visible in Fig. 1. Investigating at such wavelengths are helpful to check the feasibility of the sensor to detect nominal changes in lactate absorbance.

Simulations were carried out at *three* characterizing source-detector separations that are typically used in the commercial spectrophotometer fibre-optic probes (i.e., 0.7 mm, 1 mm and 1.5 mm).

The anatomical details of a finger-ROI were discussed in our previous publication^34^. A similar model has been used in this work. The semi-infinite tissue-volume had a width and a thickness each of 1.3 cm. The dimension was approximated based on the average measured thickness and width of the index-fingers (phalanx area) of the human volunteers who participated in our previous pilot in-vivo study^34^. The measurement was carried out on eight (5 male and 3 female) healthy human volunteers with age ranging from 20 to 35 years. Necessary ethical approval was gained from the Senate Research Ethics Committee at City, University of London and written informed consent was sought from all the volunteers prior to the commencing of the study. The experiment took place in the Physiological Measurement laboratory of the biomedical engineering research centre at City, University of London. All experiments were performed in accordance with relevant guidelines and regulations.

In the tissue volume, the stratum corneum and epidermis, the two outermost skin layers, were bloodless, and water made up 5% and 20% of those tissue volumes (including blood plasma), respectively. Dermis was divided into four sublayers depending on the different distributions in blood content at different depths: papillary dermis (0.1 mm thick, 4% blood, 50% water), upper blood net dermis (0.08 mm thick, 30% blood, 60% water), reticular dermis (0.2 mm thick, 4% blood, 70% water), and deep blood net dermis (0.3 mm thick, 10% blood, 70% water). Normal and hyperlactatemic physiological conditions were simulated by varying the blood-lactate concentrations through a range of 1–6 mmol/L.

The tissue model was optically characterized at the SWIR wavelengths by—(a) the volumetric distributions of the absorbers (i.e., epidermal melanin, blood lactate, lipid, and water), and (b) the *Rayleigh* and *Mie* scattering distributions by the small and large scale scatterers (i.e., dermal collagen and epidermal keratin). The details of the wavelength-dependent optical parameters (e.g., absorption coefficient $\mu _a$ and scattering coefficient $\mu _s$) are illustrated in Table 1. The lactate and water absorption coefficients were deduced from the optical spectra recorded in our lab and the rest of the parameters were adapted from published literature^35–39^. The anisotropy factor ($g=0.9$) and refractive index ($n=1.4$) were considered the same at all wavelengths as the variations in such parameters do not influence the model outcomes^36^.

**Table 1:** Optical properties at SWIR: the absorption coefficients of water ($\mu _{a_w}$), lactate ($\mu _{a_{lact}}$), lipid ($\mu _{a_{lip}}$), melanin ($\mu _{a_{mel}}$); and the scattering coefficients of skin ($\mu _{s_{skin}}$) and hypodermal fat ($\mu _{s_{fat}}$) are illustrated at the characterising wavelengths ($\lambda$).

$\lambda (\text{nm})$	$\mu _{a_w}~(\text{mm}^{-1})$	$\mu _{a_{lact}}~(\text{mm}^{-1})$	$\mu _{a_{lip}}~(\text{mm}^{-1})$	$\mu _{a_{mel}}~(\text{mm}^{-1})$	$\mu _{s_{skin}}~(\text{mm}^{-1})$	$\mu _{a_{fat}}~(\text{mm}^{-1})$
1310	0.12	0.29	0.18	2.75	4.9	10.57
1550	1.07	0.33	0.17	1.57	3.62	9.63
1650	0.48	0.35	0.15	1.27	3.25	9.47
1684	0.45	0.37	0.21	1.19	3.14	9.76
1730	0.61	0.37	0.42	1.09	3	13.74
1752	0.71	0.35	0.33	1.04	2.94	11.89
1920	11.45	0.33	0.5	0.77	2.52	14.63
2129	2.2	0.43	0.29	0.54	2.13	11.79
2259	1.72	0.54	0.29	0.45	1.94	15.69
2299	2.24	0.58	0.68	0.42	1.88	22.56

In the volumetric distribution consideration, the cumulative absorption coefficient $(\mu _{a_T})$ for an $i$th tissue layer, comprising volume fractions $V_b$ of blood and $V_w$ of water, at any wavelength $\lambda$ was calculated using the following equation:

1 $$\begin{aligned} \mu _{a_{T_i}} (\lambda )=V_{b_i} \cdot \mu _{a_b} (\lambda )+V_{w_i} \mu _{a_w} (\lambda ) +[1-(V_{b_i}+V_{w_i})] \cdot \mu _{a_t} (\lambda ). \end{aligned}$$

Considering the volume fractions of lipids and lactate in blood as $V_{lip}$ and $V_{lact}$, respectively, the cumulative blood absorption coefficient $\mu _{a_b}$ was calculated using the following equation:

2 $$\begin{aligned} \mu _{a_b} (\lambda )=V_{lact} \cdot \mu _{a_{lact}} (\lambda )+V_{lip} \mu _{a_{lip}} (\lambda ). \end{aligned}$$

In above equations, $\mu _{a_w}~,~\mu _{a_{lact}}~,~\mu _{a_{lip}}~$ are the absorption coefficients of water, lactate, and lipid, respectively. The baseline tissue absorption coefficient (i.e., tissue with no water, blood, or any other absorbers) was calculated following equation:

3 $$\begin{aligned} \mu _{a_t} (\lambda )=7.84 \times 10^8 \times \lambda ^{-3.255}. \end{aligned}$$

The simulated optical source generated Gaussian beam of radius of 0.1 mm, and the circular detector was of 0.2 mm radius. In each iteration, a large number of photon packets $(10^9~-~10^{10})$ were computed. MATLAB (Mathworks, Inc. USA) platform was chosen for coding and a multi-thread programming environment was used for facilitating the simulation.

## Results

**Figure 3:**
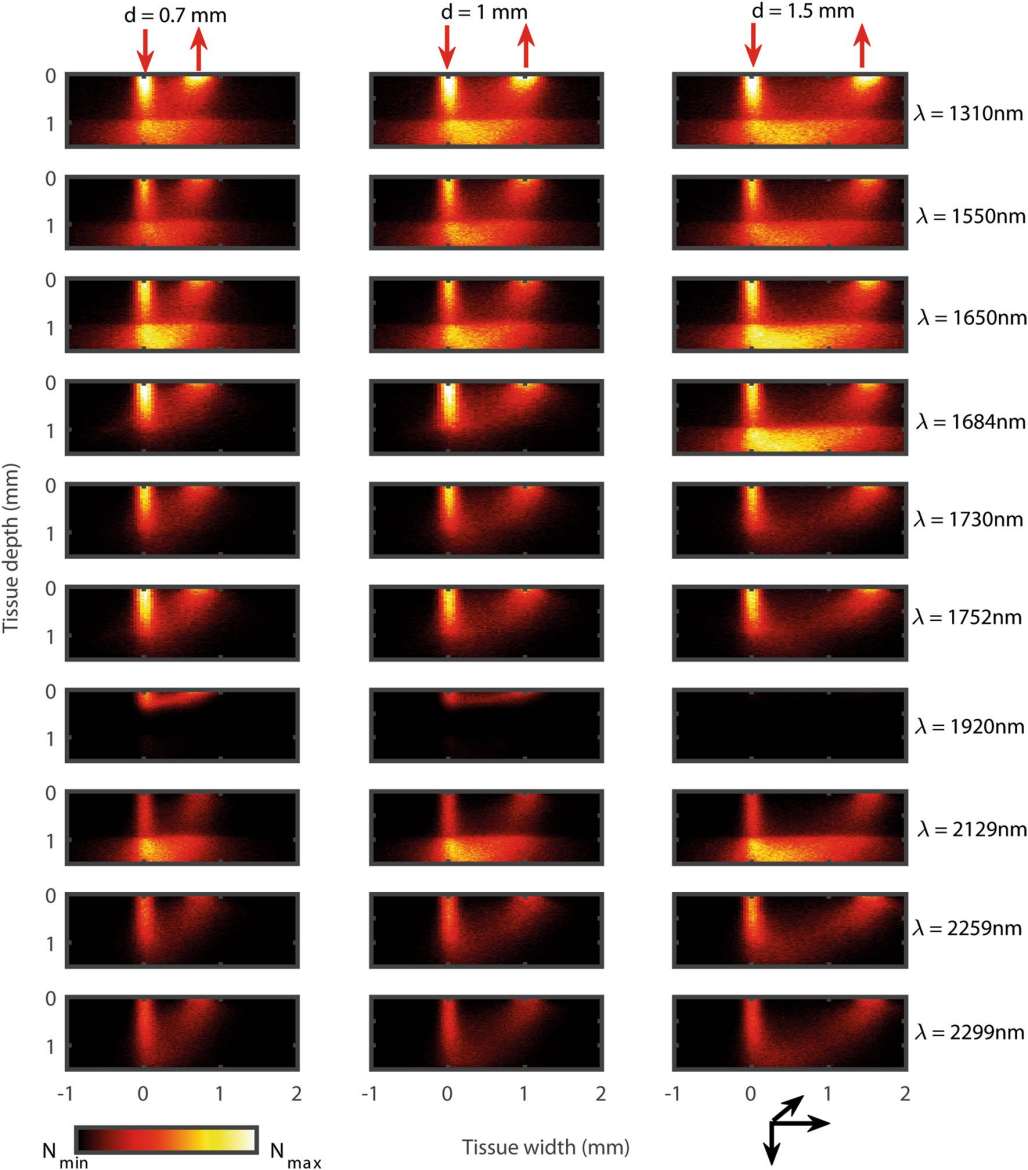
Monte Carlo simulated light-tissue interaction profiles. The results show the distribution of the light-tissue interaction events in a reflectance mode optical sensor geometry having a source (downward red arrow) and a detector (upward red arrow). Results at three source-detector separations ($d~=~0.7 \, \text{mm},~1\, \text{mm}~and~1.5\, \text{mm}$) and the ten SWIR wavelengths ($\lambda ~=~$1310 nm, 1550 nm, 1650 nm, 1684 nm, 1730 nm, 1752 nm, 1920 nm, 2129 nm, 2259 nm and 2299 nm) are presented along the columns and the rows, respectively. The colorbar represents the number distribution of the interaction events (N) between its minimum and maximum values.

The distribution of the light-tissue interaction events (absorption + scattering) through the dermal and subdermal tissue layers of the finger ROI at different optical sensor geometries are illustrated in Fig. 3.

The light-tissue interaction profiles vary significantly for different combinations of wavelengths and source-detector separations. Light does not penetrate beyond the hypodermal fat ($\le ~1.5\, \text{mm}$) at any combination of the sensor specifications. At 1920 nm, very few photons are able to penetrate through the tissue-layers because of the very high absorbance at this wavelength. At all simulated wavelengths, light penetrates through the outermost bloodless skin tissue layers, i.e., stratum corneum and epidermis, and reach the vascularized dermis. The scattering coefficient of the skin collagen reduces exponentially with increasing SWIR wavelength, resulting in lower probability of multiple scattering in the medium^40^, hence, fewer light-tissue interaction events through the skin. Scattering in the hypodermis is governed by the lipid droplets^41^ which exhibit higher scattering compared to dermis, resulting in multiple light-tissue interaction events.

**Figure 4:**
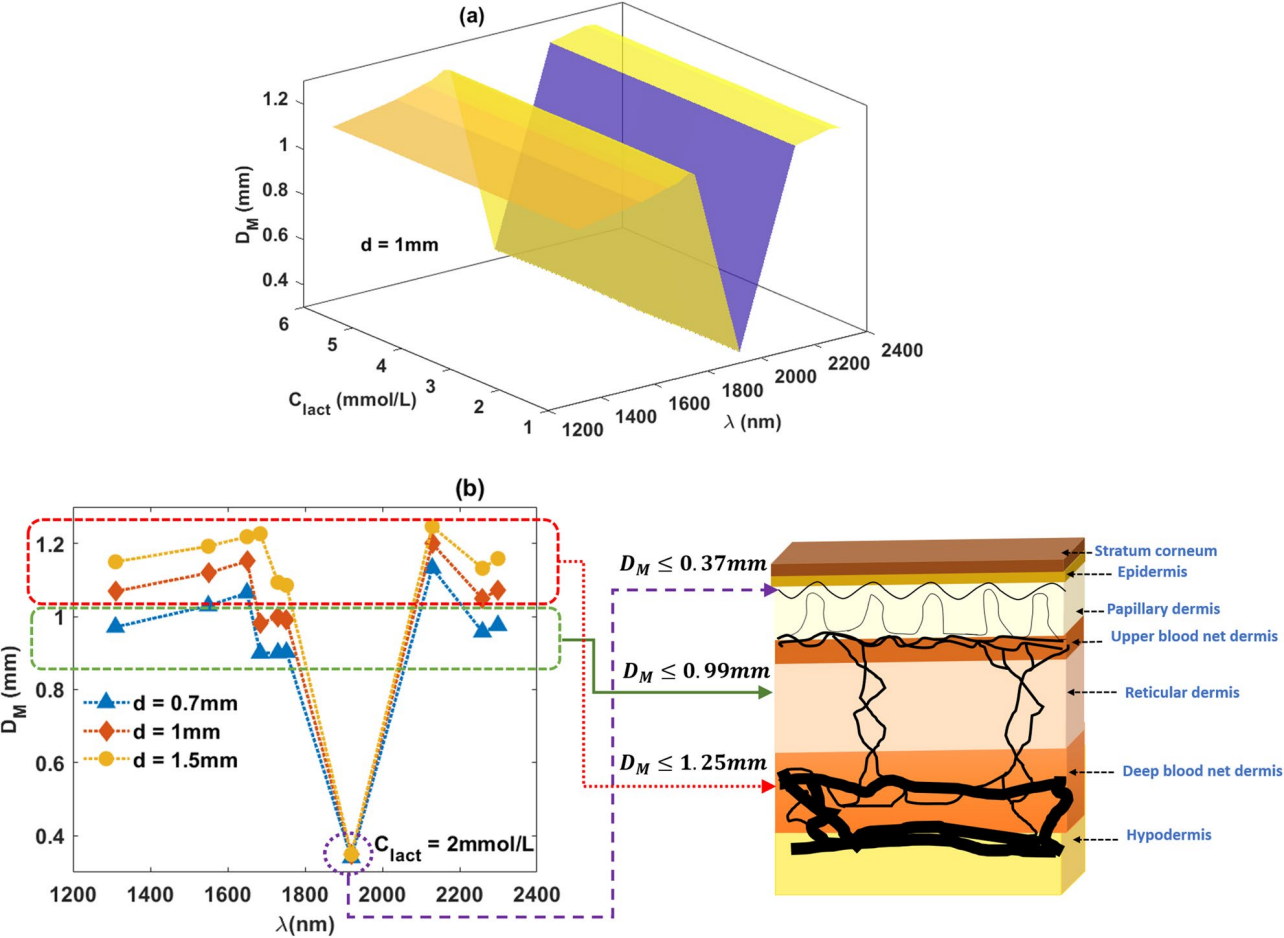
Monte Carlo simulated mean penetration depth ($D_M$) at SWIR. Simulated results at a fixed source-detector separation ($d~=~1~$mm) for the varying lactate concentrations ($C_{lact}=$ 1, 2, 3, 4, 5, and 6 mmol/L) are shown in (**a**). Simulated results for the tissue with a nominal concentration of blood lactate ($C_{lact}=$ 2 mmol/L) at three source-detector separations ($d=$ 0.7 mm, 1 mm, 1.5 mm) are presented in (**b**). The tissue volume interrogated at different sensor specifications are illustrated simultaneously. The magenta, green and red dotted lines correspond to three different combinations of characterizing wavelengths and source-detector separations.

The mean penetration depth ($D_M$), calculated as the mean of the maximum z-coordinate recorded through the path of each detected photon, gives an idea of the sample volume interrogated by the sensor. As shown in Fig. 4, the penetration depth varies with wavelength and remains unchanged with the varying lactate concentration. Even though the penetration depth at SWIR does not increase as significantly as seen in the near-infrared region, still a gradual increment is found with the increasing source-detector separation. At 1920 nm, where the water absorbance is the highest, the depth of penetration is the lowest ($D_M~\le ~0.37$ mm; within papillary dermis). With a source-detector separation of 0.7 mm and at wavelengths 1310 nm, 1550 nm, 1684 nm, 1730 nm, and 1752 nm, photons travel through the moderately vascularized reticular dermis ($D_M~\le ~0.99$ mm). With the rest of the combinations of the sensor specifications, light penetrates through the deep blood net dermis ($D_M~\le ~1.23$ mm) which contains the densest vascular network.

**Figure 5:**
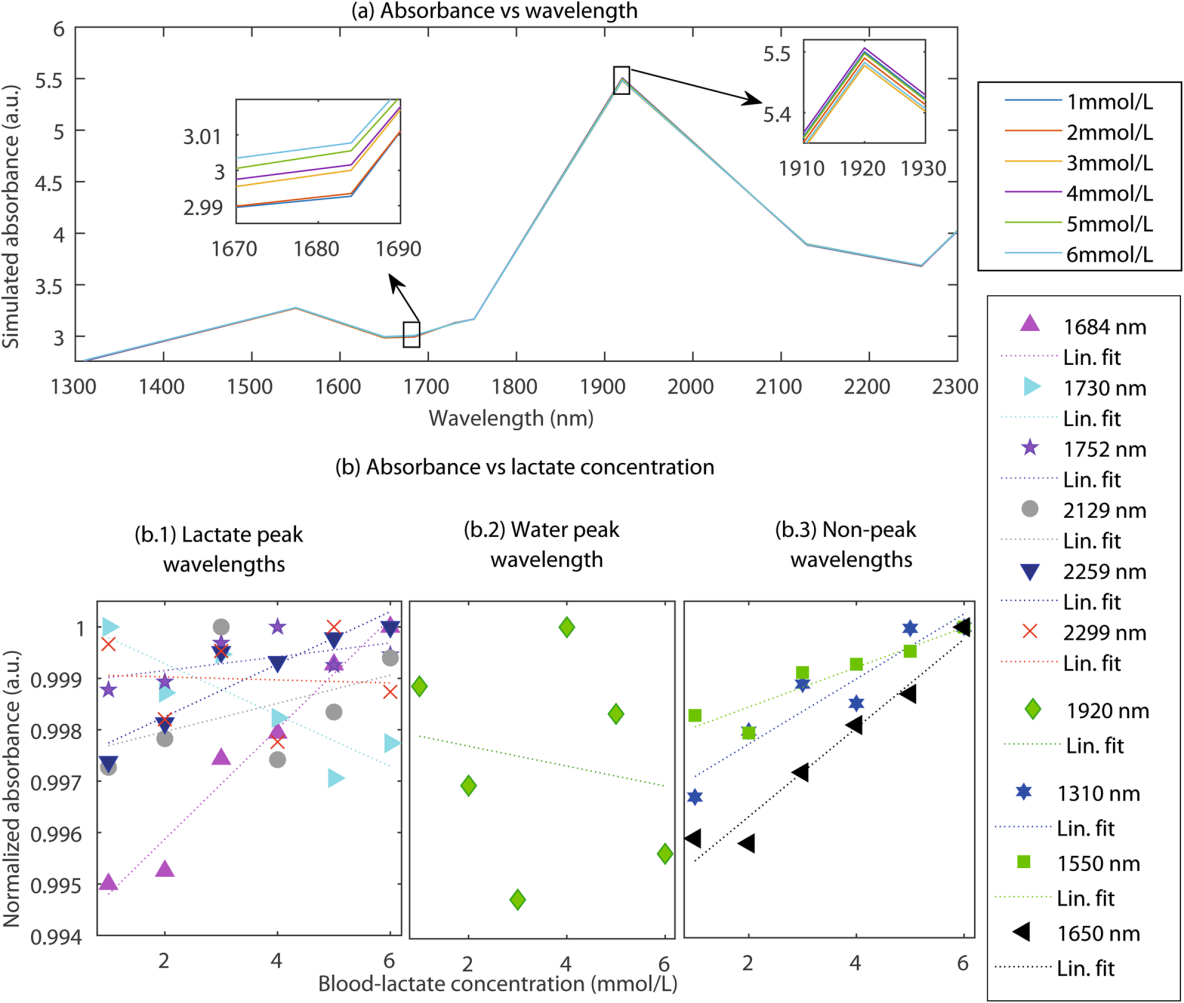
Absorbance spectra at SWIR optical wavelengths and lactate concentration. Variation in absorbance with the wavelength at $\lambda ~=~$1310 nm, 1550 nm, 1650 nm, 1684 nm, 1730 nm, 1752 nm, 1920 nm, 2259 nm and 2299 nm is demonstrated in (**a**), and the variation with the lactate concentration ($C_{lact}~=~$1–6 mmol/L) are displayed as insets. For further illustration, the wavelength sets were divided into three categories, and the variations in the normalized absorbances with lactate concentration are shown in (**b.1**) lactate peak wavelengths, (**b.2**) water peak wavelength, and (**b.3**) non-peak wavelengths. Absorbance at lactate-peak wavelengths exhibit non-linearity among the wavelengths. The water peak-wavelength exhibits random results and the non-peak wavelengths exhibit linearity. Simulations were carried out at a source-detector separation of 1 mm.

Simulated absorbance variation with wavelength and lactate concentration is presented in Fig. 5a. The simulated absorbance spectra resembles the water and lactate absorbance distribution shown in Fig. 1. The simulated absorbance value ranges between 0–6 a.u. which is similar to the experimentally acquired spectra. Also, the absorbance peaks present at 1550 nm and 1920 nm wavelengths are similar to the experimental spectra. The observational discrepancy between Figs. 1a and 5a is due to the simulation at the discrete wavelengths rather than the continuous wavelengths. With variations in lactate concentration, small but detectable changes in absorbance are found. Simulated absorbance was comparable to the results published by Maruo et. al.^42^ and agree with our previous in-vitro study^5^.

Further processing of the data revealed different trends of the absorbance variation at different wavelengths. As shown in Fig. 5b, the wavelengths were categorized in three sections: (b.1) lactate-peak wavelengths (1684 nm, 1730 nm, 1752 nm, 2129 nm, 2259 nm, and 2299 nm), (b.2) water peak wavelength (1920 nm), and (b.3) non-peak wavelengths (1310 nm, 1550 nm, and 1650 nm). The categories were chosen based on the absorbance peaks shown in Fig. 1a. The wavelength 1684 nm is shown to be the most sensitive to the lactate concentration variation. At the rest of the lactate-peak wavelengths, absorbance varies slowly or decreases with the lactate concentration which attributes to the prevailing lipid concentrations at those wavelengths. At the water peak-wavelengths, absorbance is random and not sensitive to the lactate concentrations. At the non-peak wavelengths, though no prominent peaks of lactate or water exists, the overall absorbance of lactate is higher compared to other absorbers such as water and lipid, resulting in a linear increase in absorbance with lactate concentration.

The relative power was calculated as a product of the relative detected intensity and the effective cross-sectional area of the detector. As seen in Table 2, the maximum power measured at the output of the sensor is 2.51% of the incident power. Output power significantly decreases at higher source-detector separations, for example, at 1684 nm, the relative output power at 0.7 mm, 1 mm and 1.5 mm separations are 1.28%, 0.81% and 0.55%, respectively. The overall power consumption is higher at wavelengths beyond 1752 nm, with the minimum at 1920 nm.

**Table 2:** Relative output power at different sensor specifications.

Relative detected power (%)			
$\lambda ~(\text{nm})$	$d = 0.7\, \text{mm}$	$d=1 \, \text{mm}$	$d=1.5 \, \text{mm}$
1310	2.51	1.74	1.04
1550	0.9	0.53	0.26
1650	1.57	1.03	0.57
1684	1.28	0.81	0.55
1730	0.91	0.52	0.28
1752	0.89	0.52	0.28
1920	1.67E−02	3.17E−03	3.86E−04
2129	0.27	0.13	4.80E−02
2259	0.35	0.17	8.06E−02
2299	0.19	8.03E−02	0.0284

## Discussion

A robust three-dimensional Monte Carlo model of vascular tissue-volume of an ROI (finger) has been explored for the first time at SWIR wavelengths. Though a few past studies investigated the mid-infrared optical interactions with tissue using MC models^9,10,12^, none of them included lactate optical properties in the blood. The current model incorporated the variance reduction techniques and multi-threading approach for a faster computation which enabled the simulation even at very high water-absorbing wavelengths such as 1920 nm. The average computation time was 7 h which may be improved using GPU-accelerated methods in the future.

A careful choice of the source-detector separation and the operating wavelength is of utmost importance to detect the small-scale absorbance changes due to lactate in presence of the very strong water absorbance at the SWIR wavelength range. The MC results presented in this paper have been utilized to optimize the source-detector separation and the wavelength of a non-invasive optical sensor of lactate based on the three criteria as follow:

- *Reduced noise:* input light signal penetrates sufficiently deep to reach the vascular regions of skin, confirming the signal is coming from the blood-lactate and not the bloodless sublayers of skin such as stratum corneum or epidermis, in which case it would result in noise;
- *High sensitivity:* it enables capturing small changes in lactate concentration in blood;
- *Optimal power level:* the relative output power of the sensor is sufficiently high.

The sensor design optimisation, therefore, has been carried out based on the qualitative and quantitative analysis on the $10\times 3$ combinations of the sensor specifications. For example, light penetrates through the densest vasculature (deep blood net dermis) at a source-detector separation of 1.5 mm and wavelength of 2129 nm, making it a justified choice according to the first aforementioned criteria. However, the further the light penetrates through tissue, the higher is the power consumption. Consecutively, the output detected power at this combination of sensor specifications is as low as $0.0048\%$ which does not satisfy the third aforementioned criteria.

After a careful assessment on the datasets, we conclude that the combination of $\lambda =1684$ nm, $d=1$ mm are the most feasible choice for the sensor design. At 1 mm separation distance, light penetrates through the well-perfused reticular dermis region, therefore, a noise-free signal can be acquired. The wavelength 1684 nm is the most sensitive to the lactate concentration changes in blood. The output power at this combination of specifications is approximately 1% of the input power which can be achieved utilizing the standard fiber-optic spectrophotometer probes^43^. Also, modern age *Ge* and *InGaAs* photodiodes having high responsivity at the SWIR wavelengths can be implemented in the compact wearable design of the sensor^44^.

Based on the information obtained from the current in-silico study, an integrated optical sensor will be developed utilising our lab facilities and will be used for future in-vivo studies. In the planned in-vivo investigation on healthy volunteers, in order to obtain optical spectra at different lactate levels in blood, their stress levels will be increased within a permitted range, inducing lactate formation. The volunteers will be asked to engage in controlled physical activity in regular intervals. In each interval, the lactate level of their blood will be measured by collecting sample using the commercial invasive lactate-testing kit. Simultaneously the optical spectra will be recorded from their finger utilising the sensor prototype designed utilising the optimised simulated specifications. After each interval, the resistance level of the physical activity will be increased, causing higher lactate concentration in body, and a similar protocol will be repeated. Each step of the experiment would be executed in the presence of a clinical expert and upon verbal and written consents from the volunteers. At the end of the study, lactate absorbance spectra will be plotted against different lactate concentrations and will be compared with the in-vitro data. We hope, with our systematic and combined approaches, we will be able to develop a non-invasive optical sensor for lactate detection which will be sent out for the clinical trial.

A real-time detection of blood-lactate concentration appears to be challenging because of the high water absorbance and low signal strength. The mixed trends found at different SWIR wavelengths with the lactate concentration variation indicate a non-linear relationship between the variables. Thus, the calibration of the sensor cannot be achieved through any standard regression analysis, and supervised machine learning algorithms must be incorporated. In our following in-vivo experiments, to extract lactate signature from the spectra in the presence of other absorbers such as water, sodium, and potassium chloride, and predict the lactate concentration, the Partial Least Squares (PLS) regression analysis will be implemented. The PLS model will be validated using Leave-One-Out cross-validation (LOOCV) routine to measure how accurately the model performs in practice. Such a sophisticated algorithm has been already developed to analyse our preliminary in-vitro data set^4,5^ which will be improved and applied on the in-vivo data in future.

## Conclusion

Monte Carlo model of light-tissue interactions at the SWIR wavelengths has been explored to investigate the spectroscopic signature of blood-lactate. Simulated studies showed the details of light-tissue interactions at those wavelengths and optimized a source-detector separation of 1 mm and a wavelength of 1684 nm for the sensor design. The information obtained from our in-silico investigation will be utilized for the future development of a novel integrated optical sensor for blood-lactate monitoring which will be evaluated through the in-vivo experiments and machine learning based data analysis. Upon the successful completion of our in-silico, in-vitro and in-vivo studies, the developed and standardized sensing system will be used for non-invasive real-time lactate measurements in both clinical and homecare settings.
